# SPARC and GluA1-Containing AMPA Receptors Promote Neuronal Health Following CNS Injury

**DOI:** 10.3389/fncel.2018.00022

**Published:** 2018-02-01

**Authors:** Emma V. Jones, Yann Bernardinelli, Juan G. Zarruk, Sabrina Chierzi, Keith K. Murai

**Affiliations:** ^1^Centre for Research in Neuroscience, Department of Neurology and Neurosurgery, Brain Repair and Integrative Neuroscience Program, The Research Institute of the McGill University Health Centre, Montreal General Hospital, Montreal, QC, Canada; ^2^Neonomia, Geneva, Switzerland

**Keywords:** astrocyte, synapse, glutamate receptor, matricellular protein, injury, neuroprotection, SPARC

## Abstract

The proper formation and maintenance of functional synapses in the central nervous system (CNS) requires communication between neurons and astrocytes and the ability of astrocytes to release neuromodulatory molecules. Previously, we described a novel role for the astrocyte-secreted matricellular protein SPARC (Secreted Protein, Acidic and Rich in Cysteine) in regulating α-amino-3-hydroxy-5-methyl-4-isoxazolepropionic acid receptors (AMPARs) and plasticity at developing synapses. SPARC is highly expressed by astrocytes and microglia during CNS development but its level is reduced in adulthood. Interestingly, SPARC has been shown to be upregulated in CNS injury and disease. However, the role of SPARC upregulation in these contexts is not fully understood. In this study, we investigated the effect of chronic SPARC administration on glutamate receptors on mature hippocampal neuron cultures and following CNS injury. We found that SPARC treatment increased the number of GluA1-containing AMPARs at synapses and enhanced synaptic function. Furthermore, we determined that the increase in synaptic strength induced by SPARC could be inhibited by Philanthotoxin-433, a blocker of homomeric GluA1-containing AMPARs. We then investigated the effect of SPARC treatment on neuronal health in an injury context where SPARC expression is upregulated. We found that SPARC levels are increased in astrocytes and microglia following middle cerebral artery occlusion (MCAO) *in vivo* and oxygen-glucose deprivation (OGD) *in vitro*. Remarkably, chronic pre-treatment with SPARC prevented OGD-induced loss of synaptic GluA1. Furthermore, SPARC treatment reduced neuronal death through Philanthotoxin-433 sensitive GluA1 receptors. Taken together, this study suggests a novel role for SPARC and GluA1 in promoting neuronal health and recovery following CNS damage.

## Introduction

Astrocytes communicate with neurons through secretion of active molecules to control synapse development, neuronal activity and plasticity (Clarke and Barres, 2013; Baldwin and Eroglu, 2017). Upon stress caused by injury or disease, astrocytes respond by becoming ‘reactive’ by changing their morphology and gene expression and, together with microglia, respond to the damage and actively participate in recovery of the tissue (Sofroniew, 2009; Sofroniew and Vinters, 2010). One mechanism utilized by reactive astrocytes is the expression of secreted factors which promote tissue and ECM remodeling around injury sites, such as matricellular proteins.

Several recent studies have drawn attention to the significant roles that matricellular proteins play in central nervous system (CNS) development and disease. Matricellular proteins can be defined as secreted, non-structural regulators of the extracellular matrix and cell-cell interactions which act through modulation of growth factor, adhesion, cytokine and protease signaling (Bornstein and Sage, 2002; Frangogiannis, 2012). Many of the matricellular proteins in the CNS, such as SPARC, Hevin/SC1 (SPARC-like 1), Thrombospondins, Glypicans, CYR61/Connective Tissue Growth Factor/Nov family of proteins (CCN) and Tenascin C, are secreted from glial cells (Eroglu, 2009; Jones and Bouvier, 2014). Although these molecules are structurally unrelated, they share the general characteristic that they are highly expressed during development then become downregulated to a low level in the mature CNS. However, upon injury or disease, their expression has been shown to be upregulated, where they are well-positioned to contribute to repair processes such as tissue remodeling, proliferation, angiogenesis, and rewiring of neural circuitry (Jones and Bouvier, 2014).

In our previous study we examined the role of SPARC during development and found that SPARC regulates the levels of surface α-amino-3-hydroxy-5-methyl-4-isoxazolepropionic acid receptors (AMPARs) during synapse maturation, which in turns modulates synaptic strength and plasticity (Jones et al., 2011). Furthermore, we found that SPARC levels were dynamically regulated by neural activity. In the mature nervous system, SPARC has been shown to be upregulated following injury or disease (reviewed in Jones and Bouvier, 2014; Jayakumar et al., 2017) but its role is not yet fully understood. Here we investigate the expression of SPARC following ischemic insult *in vitro* and *in vivo*. Using oxygen and glucose deprivation (OGD) to simulate ischemia in hippocampal slices *in vitro*, we uncovered a novel role for SPARC in regulating AMPAR and promoting neuroprotection following CNS injury.

## Materials and Methods

### Animals and Recombinant SPARC Protein

WT C57BL/6J mice were used for all experiments. Animal procedures were performed in accordance with the guidelines of the Canadian Council for Animal Care and the Montreal General Hospital Facility Animal Care Committee.

Recombinant SPARC protein was purchased from R&D Systems (Mouse; Cat no. 942-SP-050). In all experiments, a concentration of 0.5 μg/ml SPARC was used. This concentration was determined by titration of different SPARC concentrations and analysis of its effect on surface GluA1 levels (Supplementary Figure 1).

### Dissociated Hippocampal Cultures

Dissociated mouse hippocampal neurons were grown suspended above astrocyte feeder cultures using a method modified from Kaech and Banker, 2006 published previously (described in detail in Jones et al., 2012). An astrocyte feeder layer was prepared by plating mouse astrocytes at 20,000 cells/cm^2^ onto 12-well dishes coated with poly-D-lysine (0.1 mg/ml) (Sigma) in Minimal Essential Medium containing Earle’s salts and L-glutamine supplemented with 10% horse serum, 0.6% glucose, and 1% penicillin/streptomycin (Invitrogen). Media was replaced with Neurobasal-A medium supplemented with 2% B27, 1 mM GlutaMax, and 1% penicillin/streptomycin (Invitrogen) 24 h before neuron dissection. After 5 days of growth of astrocyte cultures, hippocampal neurons were isolated from P0 pups. Hippocampi were dissociated by treatment with papain (0.1% papain, 0.02% BSA in Neurobasal-A medium, 15 min at 37°C) followed by trituration with a fire-polished glass pipette in Neurobasal-A media containing trypsin inhibitor (1%, Sigma) and BSA (1%). Neurons were plated onto poly-L-lysine-coated coverslips (0.1 mg/ml Sigma) at a density of 20,000 cells/cm^2^ for a period of 3 h before transfer to dishes containing the astrocyte feeder layers. Coverslips were suspended above the feeder layer on wax dots adhered to the bottom of the culture dish well.

### Immunofluorescence (IF) Labeling of Dissociated Neurons

We used a protocol described previously (Stellwagen et al., 2005; Jones et al., 2011) to visualize surface GluA1 and GluA2-containing AMPARs under non-permeabilizing conditions. Briefly, 14-day-old cultures were washed with ice-cold PBS then fixed with 4% paraformaldehyde/0.1 M phosphate buffer for 10 min on ice. The coverslips were blocked in 5% BSA and incubated with an antibody against the N terminus of GluA1 (Millipore clone RH95 1:30) and GluA2 (Millipore clone 6C4 1:200) for 1 h. To analyze surface GluA1 and GluA2 levels, blinded images were thresholded using ImageJ (NIH) to exclude background noise and values were held constant across each experiment. For each neuron, we measured a 50 μm region of three dendrites (at least one body distance away). The average pixel intensity was recorded for each dendrite (>70 measured per condition for each experiment) and used to compare surface GluA1 and GluA2 levels across conditions.

### Cell Surface Biotinylation

To examine the surface expression of AMPAR subunits GluA1 and GluA2, neurons were gently washed twice with ice-cold PBS (containing Ca^2+^ and Mg^2+^) and then incubated with Sulfo-NHS-SS-Biotin/PBS (0.2 mg/ml) for 30 min at 4°C. Cells were then washed twice with 100 mM glycine/PBS to quench the biotin reaction, followed by lysis on ice with PBS/0.1% Triton X-100/0.1% SDS supplemented with protease inhibitors and sodium orthovanadate. Protein levels were assessed by BCA assay and normalized with lysis buffer to ensure equal input into the immunoprecipitation. An aliquot of lysate was kept for analysis of total receptor levels and the remainder was incubated with streptavidin beads (Sigma) for 2 h at 4°C on a rotating platform. Biotinylated proteins were eluted with 3X sample buffer, resolved by SDS-PAGE and analyzed by immunoblotting with GluA1 (Millipore clone RH95; 1:9000) and GluA2 (Millipore clone 6C4; 1:2000) antibodies. Immunoblotting with a GAPDH antibody demonstrated that only cell surface proteins were isolated with this method. Densitometric quantification was carried using ImageJ (NIH). Cell surface receptor values were adjusted relative to total receptor levels (GluA1/GluA2).

### Organotypic Hippocampal Slice Cultures, Synaptosome Preparation, and Western Blotting

Organotypic hippocampal slices were prepared as described previously (Stoppini et al., 1991; Murai et al., 2003; Zhou et al., 2007). Briefly, 300 μm slices from postnatal day 6–7 mouse pups were made with an automatic tissue chopper and transferred onto semiporous tissue culture inserts (0.4 μm pore size; Millipore) containing media [50% minimum essential medium, 25% horse serum, 25% HBSS, 6.5 mg/ml D-glucose, and 0.5% penicillin/streptomycin (Invitrogen)]. Medium was replaced three times per week. For immunofluorescence (IF) labeling slices were fixed in 4% paraformaldehyde/0.1M phosphate buffer for 30 min, washed with Tris-buffer saline (TBS) and blocked for 1 h at room temperature in 10% donkey serum (Jackson Immunoresearch Laboratories) in TBS containing 0.2% Triton X-100 (TBS-T) and then incubated with a goat anti-SPARC antibody (R&D Systems AF942 1:300) overnight. The next day, slices were washed four times with TBS-T before incubation with donkey anti-goat Alexa-568 (1:1000 Molecular Probes) for 1 h at room temperature. Slices were then washed four times with TBS-T and imaged with confocal microscopy. For synaptosomal preparations, slices were lysed in Syn-PER Synaptic Protein Extraction Reagent Thermo Scientific #87793, characterized in Palavicini et al., 2013; Wang et al., 2016 and in Cook et al., 2014. The synaptosomal fraction was extracted as per supplier’s directions. Briefly, slices were scraped into 200 μl of Syn-PER containing protease inhibitors and dounced using a glass homogenizer. The homogenate was centrifuged at 1200 *g* for 10 min at 4°C to remove cell debris. The supernatant (‘crude’ fraction) was then centrifuged for 20 min at 15,000 *g* at 4°C. The resulting supernatant was removed, and the pellet (‘synaptic’ fraction) was resuspended in Syn-PER reagent. The protein concentration of each fraction was determined by BCA assay. The crude and synaptic fractions were diluted in 3X sample buffer, resolved by SDS-PAGE, and analyzed by immunoblotting with the following synaptic antibodies: mouse anti-GluA1 (Millipore clone RH95; 1:9000), mouse anti-GluA2 (Millipore clone 6C4; 1:2000), mouse anti-vGlut1 (Neuromab clone N28/9; 1:7000), mouse anti-PSD-95 (Neuromab clone K28/43; 1:300,000), mouse anti-GluN1 (BD Pharmingen; 1:3000) and mouse anti-GAPDH (loading control; Millipore clone 6C5 1:100,000). For other immunoblot analysis, slices were lysed on ice in Triton lysis buffer (20 mM Tris pH 7.4, 137 mM NaCl, 25 mM beta-glycerophosphate, 2mM EDTA, 1% Triton X-100, 10% glycerol, and 0.1% SDS supplemented with protease inhibitors and sodium orthovanadate). Lysates were centrifuged at 13,000 rpm for 10 min at 4°C to pellet cell debris. Supernatants were diluted with 3X sample buffer, resolved by SDS-PAGE, and analyzed by immunoblotting with antibodies anti-SPARC (R&D Systems AF942; 1:3000) and GAPDH as a control for protein levels.

### GluA1/GluA2 AMPA Receptor Complex Analysis

Following synaptosomal purification, we carried out co-immunoprecipitation experiments for GluA1 and GluA2 using a previously published protocol (Kang et al., 2012; Cook et al., 2014). The immunoprecipitated AMPAR complexes were diluted in 3X sample buffer, resolved by SDS-PAGE and analyzed by immunoblotting using GluA1 and GluA2 antibodies (as above). The unbound supernatant fraction from the co-immunoprecipitation was blotted for GAPDH to ensure equal protein loading.

### mEPSC Recordings

Miniature excitatory postsynaptic current (mEPSCs) were recorded by whole-cell patch recordings made on CA1 pyramidal cells from mouse organotypic hippocampal slices after 13–19 DIV. Slice cultures were maintained at 32°C in a carbogenated (5% CO_2_/95% O_2_) interface chamber under artificial cerebrospinal fluid (ACSF) perfusion. Recordings were made with an Axopatch 200B (Molecular Devices) using low-resistance pipettes (2–5 MΩ) containing 140 mM K-gluconate, 5 mM NaCl, 2 mM MgCl_2_, 0.1 mM CaCl_2_, 1.1 mM EGTA, 7 mM Na_2_-phosphocreatine, 10 mM HEPES, 4 mM Mg-ATP, and 0.4 mM Na_3_-GTP. Cells were held at -65 mV with 1 μM TTX and 50 μM picrotoxin. Membrane currents were monitored in voltage-clamp mode using pClamp software (Molecular Devices). Series resistance was compensated and checked before and after every recording period; cells containing >20% resistance change were excluded from the analysis. mEPSCs were collected over a 5–10 min period from control or 48h SPARC-treated hippocampal slices (0.5 μg/ml), with or without Philanthotoxin 433 (5 μM). Custom written software (Courtesy Pablo Mendez, University of Geneva, Medical School, Switzerland) was used for analyzing mEPSC events. Briefly, individual events were detected with a threshold-shape process. Detection criteria based on threshold was adjusted to ignore slow membrane fluctuations and electric noise. Events smaller than -4 pA were discarded. To obtain the cumulative mEPSC amplitude, all event amplitudes were collected for each cell recording. An average amplitude was then calculated by cumulating the first 100 events from all experiments. The mean mEPSC frequency was obtained by dividing the number of event by the duration of a given recording. An average frequency was then calculated across experiments. All data are presented as mean ± SEM, and comparisons were made using ANOVA with *post hoc* Holm–Sidak test as specified. Differences were considered to be statistically significant ^∗∗∗^*p* < 0.001.

### Induction of Focal Cerebral Ischemia Using Middle Cerebral Artery Occlusion (MCAO)

Surgery leading to focal cerebral ischemia was conducted as described previously (Zarruk et al., 2012) and a variant of a model described earlier (Chen et al., 1986; Liu et al., 1989). In brief, animals were put under anesthesia with isoflurane in O_2_ (0.5 L/min) during the whole procedure. During surgery, body temperature was maintained at 37.0 ± 0.5°C using a homeothermic system with a rectal probe (Harvard apparatus). A small craniotomy was made over the trunk of the left middle cerebral artery (MCA) and above the rhinal fissure. The permanent middle cerebral artery occlusion (MCAO) was done by ligature of the trunk of the MCA just before its bifurcation between the frontal and parietal branches with a 9-0 suture. Complete interruption of blood flow was confirmed under the operating microscope. Additionally, the left common carotid artery was then occluded. Mice in which the MCA was exposed but not occluded served as sham-operated controls (sham). After surgery, animals were returned to their cages with free access to water and food. No spontaneous mortality occurred after MCAO. For IF labeling, mice were perfused with 4% paraformaldehyde and 14 μm thick coronal sections were prepared by a cryostat (Leica, CM3050 S) and processed attached to slides. After 1 h incubation in blocking solution (10% donkey serum in TBS-T), sections were incubated overnight at 4°C with the following primary antibodies: SPARC (1:300), GFAP (1:500) and IBA-1 (1:500). Slides were washed three times with TBS and primary antibodies were revealed with 2 h incubation with Alexa Fluor secondary antibodies (1:300, 10% donkey serum, TBS-T) and confocal microscopy. To assess which cell type upregulated SPARC post-MCAO, we used ImageJ (NIH) to count the total number of microglia (IBA+) and astrocytes (GFAP+) and calculate the proportion of each cell type expressing SPARC.

For immunoblotting, the cortex of the ipsilateral MCAO and sham control C57BL/6J mice was dissected at different time points (*n* = 3 mice per group) following intracardiac perfusion with PBS. Total protein was extracted with 1% Nonidet P-40 (Sigma), 1% sodium deoxycholate (BDH Chemicals), 2% SDS, 0.15 M sodium phosphate (pH 7.2), 2 mM EDTA, containing a mixture of protease inhibitors (Roche Diagnostics) as described previously (Zarruk et al., 2015). Naive animals served as controls. Samples (25 μg) were separated by SDS-PAGE gels 4-12% (Novex, life Technologies), transferred to polyvinylidene fluoride membrane (Millipore, Billerica, MA, United States).

### Oxygen-Glucose Deprivation (OGD) and Propidium Iodide (PI) Labeling

Oxygen-glucose deprivation followed by normoxic reoxyge-nation was used to simulate ischemic damage *in vitro*. Subsequent cell death was assessed by measuring cellular uptake of PI. To induce OGD we used a protocol modified from Lushnikova et al. (2004), Kawano et al. (2006) and Montero et al. (2007). A humidified modular incubator chamber (Billups-Rothenberg Inc.) and glucose-free and serum-free medium (Neurobasal A media without glucose 0050128DJ, Invitrogen), supplemented with 1 mM Sodium Pyruvate) were pre-warmed at 37°C for 30 min. The chamber was then flooded by 95% N_2_/5% CO_2_ gas for 4–6 min (to reduce the O_2_ gas level to zero), then sealed and incubated at 37°C for 15 min to deoxygenate the media. The semi-porous inserts containing the hippocampal slices (11–14 DIV) were transferred into dishes containing 1 ml of the warm deoxygenated media and washed with 0.5 ml of the media, (which was placed on top of the slices and then aspirated) before being transferred to the chamber where air was again replaced by 95% N2/5% CO_2_ for 4–6 min. The chamber was then sealed and placed in an incubator at 37°C for 45 min. Following OGD, the cultures were returned to fresh media and normoxic conditions for 24, 48, or 72 h (as indicated in the figure legend) prior to analysis. Untreated slice cultures were used as control. For experiments involving PI (Invitrogen), we added 2 μM into the slice growth media following OGD. Prior to fixation, slices were washed twice with PBS to remove excess PI and then fixed in 4% paraformaldehyde/0.1 M phosphate buffer for 30 min. Slices were permeabilised with PBS/0.2% Triton-X100 and incubated with TO-PRO-3 iodide (Invitrogen) (1:10,000) in PBS to label all cell nuclei (‘total’). The percentage of apoptotic nuclei was determined by counting the number of PI-positive nuclei relative to total number of nuclei (TO-PRO-3) using ImageJ (NIH). For these experiments, imaging was performed blindly and at least 10 images were taken per condition for each experiment.

## Results

### SPARC Induces an Increase in Synaptic GluA1-Containing AMPARs

**FIGURE 1:**
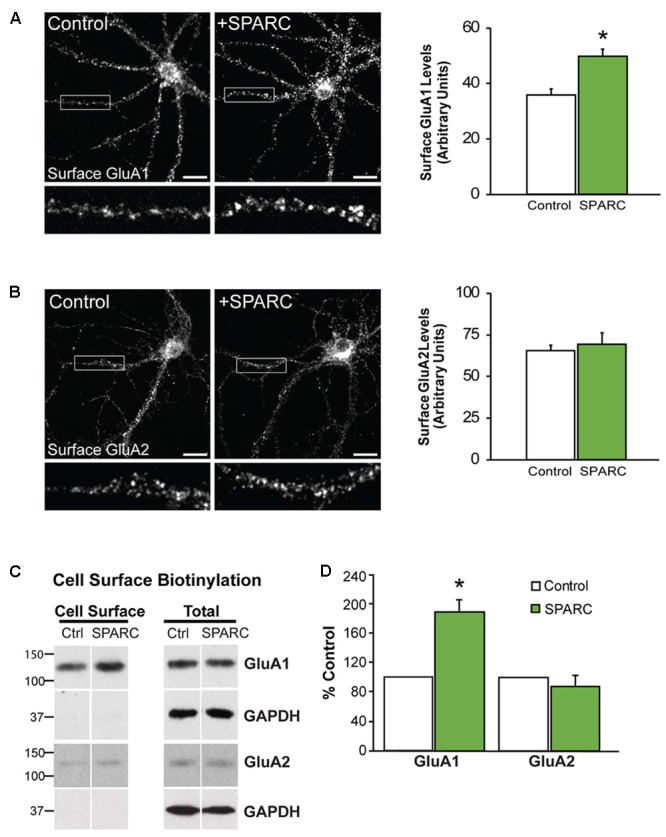
Neurons treated with SPARC have increased GluA1 but not GluA2 surface expression. Representative images of surface GluA1 **(A)** and GluA2 **(B)** expression showing increased intensity of GluA1 puncta in 14 DIV hippocampal neuron cultures treated with SPARC (0.5 μg/ml, 48 h) **(A)** but no change in GluA2 **(B)** vs. control cultures. The boxed region is magnified below each image [Student’s *t*-test, ^∗^*p* = 0.0005 (*n* = 3)]. **(C)** Representative immunoblots showing cell surface (left panel) and total GluA1 and GluA2 (right panel) levels in control cultures and cultures treated with SPARC (0.5 μg/ml, 48 h). Cell surface receptor levels were determined by cell surface biotinylation. Neurons cultured with SPARC had significantly higher surface GluA1 but not GluA2 [2-tailed, 1-sample *t*-test; *p* = 0.03, *n* = 3)] levels without an increase in total receptor levels **(D)**.

**FIGURE 2:**
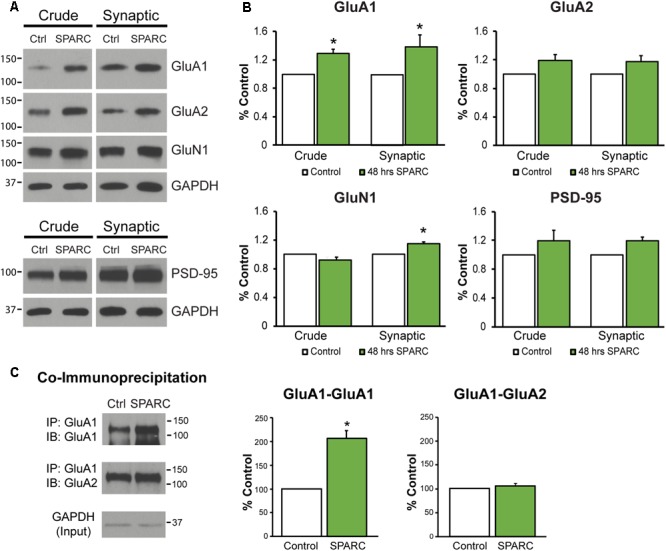
Analysis of synaptic proteins in organotypic hippocampal slices treated with SPARC reveals an increase in GluA1 levels and GluA1-containing AMPAR complexes. **(A)** Representative immunoblots showing total (left panel, ‘crude’) and synaptic (right panel) proteins (GluA1, GluA2, GluN1, and PSD-95) from synaptosome preparations of control and SPARC-treated (0.5 μg/ml, 48 h) organotypic hippocampal slices. **(B)** Hippocampal slices treated with SPARC have increased total and synaptic GluA1 [2-tailed, 1-sample *t*-test; *p* = 0.004, *n* = 4)] relative to control, but no significant change in GluA2 levels (*n* = 6). **(C)** SPARC-treated hippocampal slices show an increase in GluA1–GluA1 AMPAR complexes, whereas we did not observe a change in GluA1–GluA2 containing complexes [2-tailed, 1-sample *t*-test; *p* = 0.024, *n* = 4)].

In the developing brain, SPARC is highly expressed in CNS astrocytes and microglia, where it has been shown to regulate synapse development and maturation (Jones et al., 2011; Kucukdereli et al., 2011). Conversely, in the mature CNS, the expression of SPARC is reduced and largely confined to expression in microglia, Bergmann glia in the cerebellum and Muller glia in the retina (Vincent et al., 2008). However, there are several studies that show that following increased neuronal activity (Jones et al., 2011) or upon injury or disease, SPARC levels are upregulated (Ikemoto et al., 2000; Liu X. et al., 2005; Ozbas-Gerceker et al., 2006; Au et al., 2007; Baumann et al., 2009; Lloyd-Burton et al., 2013) to modulate neuronal behavior. Given our previous work showing that SPARC can regulate the strength of synapses during development, we decided to investigate the effect of increasing SPARC on surface AMPARs in hippocampal neuron cultures grown with a feeder layer of astrocytes. We assessed the surface levels of AMPAR subunits GluA1 and GluA2 by immunofluorescence using non-permeabilizing conditions and found that surface GluA1 but not GluA2 was significantly increased upon administration of exogeneous recombinant SPARC (48 h, 0.5 μg/ml; **Figures 1A,B**). This result was confirmed by performing surface biotinylation experiments (**Figures 1C,D**), which showed a specific increase in surface GluA1 without a change in overall levels in neurons. Next, we examined the effect of SPARC in organotypic hippocampal slices where the three-dimensional architecture of synapses and their interaction with astrocytes is preserved (Haber et al., 2006). In contrast to the dissociated neuron cultures, we found that SPARC treatment (48 h, 0.5 μg/ml) not only increased synaptic GluA1 but also total GluA1 levels. Similar to the dissociated cultures, surface and total GluA2 levels were not significantly changed. We did observe a trend for an increase in total and synaptic GluA2 levels following SPARC treatment. However, performing additional experiments did not identify a significant difference with GluA2. Overall GluN1 and PSD-95 levels were also not affected, however, we observed a small but significant increase in synaptic GluN1 levels (**Figures 2A,B**). Therefore, increasing SPARC levels induces an increase in synaptic and surface GluA1-containing AMPARs in hippocampal slices and dissociated neurons.

In the hippocampus, both GluA1-containing homomeric (GluA1-GluA1) and heteromeric (GluA1–GluA2) complexes exist, with the majority being composed of both GluA1 and GluA2 proteins (∼80%; Lu et al., 2009). Given that SPARC increased the levels of synaptic GluA1, we sought to investigate whether there was a change in the levels of GluA1-GluA1 containing AMPARs. To test this, we co-immunoprecipitated GluA1 subunits from synaptosomes (Kang et al., 2012; Cook et al., 2014) and examined the levels of associated GluA1 and GluA2 in control and SPARC-treated hippocampal slices (**Figure 2C**). We found that there was an increase in the amount of GluA1–GluA1 AMPAR interactions in SPARC treated slices compared to control, whereas GluA1–GluA2 interactions remained unaffected, suggesting that SPARC promotes interactions among GluA1 subunits in synaptic fractions.

### SPARC Increases Synaptic Function through Philanthotoxin 433 (Phx-433)-Sensitive AMPARs

**FIGURE 3:**
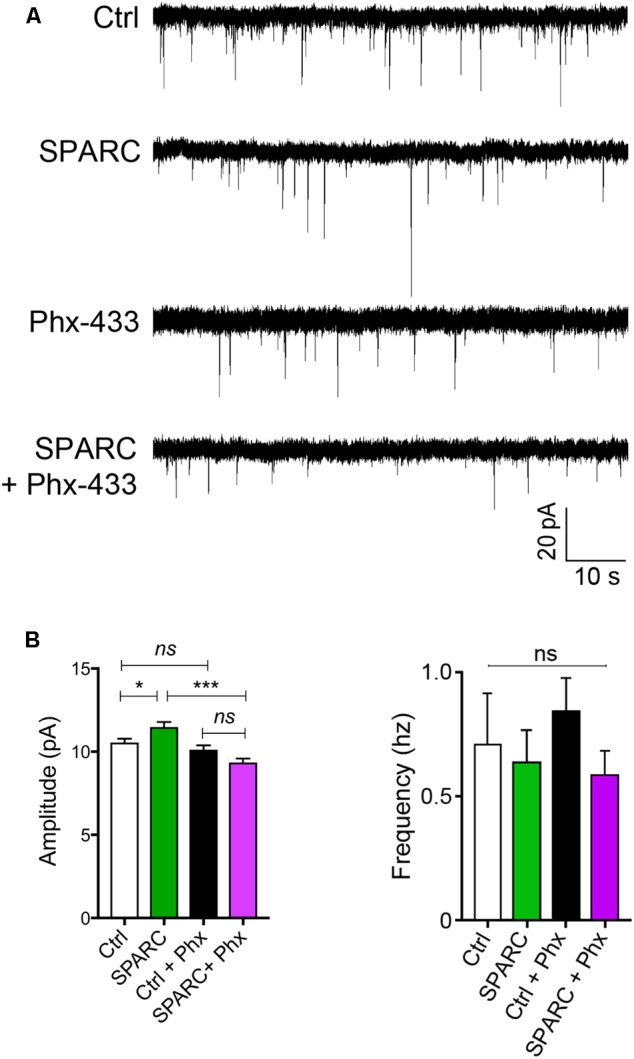
SPARC-treated organotypic hippocampal slices have increased synaptic strength which is mediated via GluA1-containing AMPARs. **(A)** Sample traces of mEPSC recordings from hippocampal neurons of control, SPARC-treated (0.5 μg/ml, 48 h), Phx-433 treated and SPARC and Phx-433 treated organotypic hippocampal slices. **(B)** Graphs of average mEPSC amplitudes (left) and frequency (right) showing significantly increased mEPSC amplitudes in hippocampal slices treated with SPARC (0.5 μg/ml, 48 h). The increase in mEPSC amplitude was inhibited when slices were also treated with Phx-433, indicating that the change in synaptic strength is mediated via GluA1-containing AMPARs [ANOVA with *post hoc* Holm–Sidak test: *n* = 10 for control, *n* = 9 for SPARC, *n* = 8 for Ctrl + Phx-433 and *n* = 8 for SPARC + Phx-433, ^∗∗∗^*p* < 0.001].

We next investigated whether the increase in GluA1 induced by SPARC affected synaptic function. To do this, we recorded AMPAR-mediated mEPSCs (miniature EPSCs) from CA1 neurons in control and SPARC-treated hippocampal slices. We found that mEPSCs in SPARC-treated slices were significantly greater in amplitude whereas their frequency was unchanged (**Figures 3A,B** and Supplementary Figures 2A,B) indicating that SPARC promotes an increase in synaptic strength. To determine whether the increase in mEPSC amplitude was due to homomeric GluA1-containing AMPAR, we treated slices with Philanthotoxin 433 (Phx-433), a polyamine-containing toxin which specifically inhibits calcium permeable AMPARs (Washburn and Dingledine, 1996), the majority of which are assumed to be GluA2-lacking, homomeric GluA1 AMPARs (Rozov et al., 2012; Mattison et al., 2014). In SPARC-treated slices, Phx-433 reduced mEPSC amplitude to baseline levels, suggesting that the increase in synaptic strength induced by SPARC was a result of additional GluA1-containing AMPARs at the synapse (**Figure 3B**). Taken together, these results suggest that SPARC induces a selective increase in functional synaptic GluA1-containing AMPARs.

### SPARC Is Upregulated in Microglia and Astrocytes Following MCAO

**FIGURE 4:**
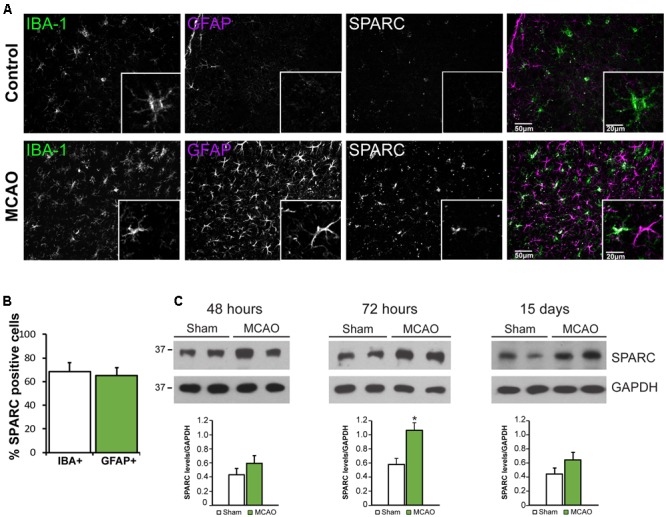
SPARC expression is upregulated in cortical microglia and astrocytes 72 h following middle cerebral artery occlusion (MCAO). **(A)** Immunofluorescence labeling for SPARC, IBA-1, and GFAP shows that SPARC is expressed predominantly in microglia in control (contralateral cortex; top panel) but is increased in both microglia and reactive astrocytes 72 h following MCAO (bottom panel) in the peri-infarct region. The peri-infarct region was defined as the region surrounding the infarct core. **(B)** SPARC is upregulated in a similar proportion of IBA+ microglia and GFAP+ astrocytes following MCAO [2-tailed, 2-sample *t*-test; *p* = 0.605, *n* = 8]. **(C)** Representative immunoblots showing changes in SPARC expression in the ipsilateral cortex at 48 h, 72 h and 15 days post MCAO compared to Sham treated animals. SPARC levels were significantly increased at 72 h post MCAO but decreased by 15 days [2-tailed, 2-sample *t-*test; *p* = 0.040, *n* = 3)].

Several studies have reported that SPARC is upregulated following challenge to the nervous system from elevated neuronal activity, glutamate exposure, or injury (Ikemoto et al., 2000; Liu Q.S. et al., 2005; Ozbas-Gerceker et al., 2006; Jones et al., 2011) including photothrombotic stroke (Lloyd-Burton et al., 2013). We chose to examine SPARC expression in the well-characterized model of ischemic cortical stroke generated by MCAO (Chen et al., 1986; Liu et al., 1989; Carmichael, 2005). MCAO produces an ischemic infarct ‘core,’ characterized by almost complete loss of neurons, surrounded by a ‘penumbra-like’ peri-infarct region, where neuronal cell death, although severe, is delayed and hence represents an important therapeutic target for potential neuroprotection and recovery (Lo, 2008; Murphy and Corbett, 2009; Ramos-Cabrer et al., 2011). Using IF labeling, we examined SPARC expression in the peri-infarct region compared to the same region in the contralateral cortex, where SPARC was present at a low level, predominantly in resting microglia (**Figure 4A**, top panel), consistent with previous studies (Vincent et al., 2008). In contrast, following MCAO, SPARC was upregulated in both microglia and reactive astrocytes (**Figure 4A**, bottom panel and **Figure 4B**). To quantify changes in SPARC expression following MCAO, we performed immunoblotting on cortical tissue dissected at 48 h, 72 h, or 15 days following MCAO or Sham surgery (**Figure 4C**). Consistent with the IF analysis, we observed a significant increase in SPARC protein levels at 72 h post MCAO, with a return to baseline levels by 15 days. We did not see any changes in SPARC in the contralateral cortex (data not shown). Thus, SPARC shows a delayed upregulation in astrocytes and microglia in response to MCAO.

### SPARC Is Increased Following OGD and Prevents the Selective Loss of Synaptic GluA1-Containing AMPARs, Leading to Improved Cell Survival of CA1 Neurons

**FIGURE 5:**
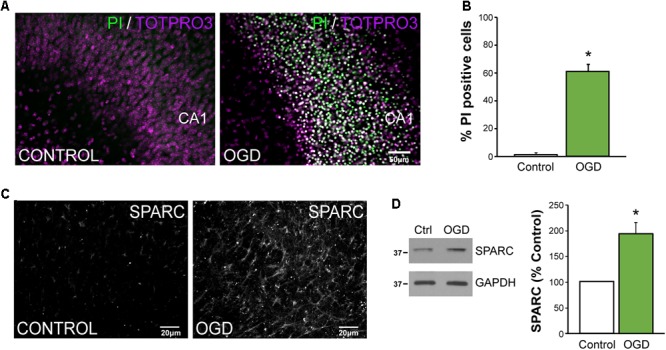
SPARC levels are elevated in an *in vitro* model of ischemic excitotoxicity **(A,B)** Organotypic hippocampal slices were exposed to oxygen and glucose deprivation (OGD) for 45 min followed by normoxic reoxgenation for 72 h. Percentage of apoptotic cells was assessed using propidium iodide (PI) labeling of apoptotic nuclei (green) relative to total cell number (TO-PRO-3 iodide; magenta). Significant death of CA1 neurons was observed following OGD [2-tailed, 2-sample *t*-test; *p* = 0.0018, *n* = 4)]. Scale = 50 μm **(C)** SPARC expression was assessed by immunofluorescence labeling in organotypic hippocampal slices at 24 h post OGD. Untreated slices of the same age were used as a control. **(D)** Representative immunoblots showing changes in SPARC expression 48 h post OGD (left panel). SPARC levels were significantly increased following OGD [2-tailed, 1-sample *t*-test; *p* = 0.0055, *n* = 4)].

To better dissect the role of SPARC after CNS injury, we used an *in vitro* model of ischemia, OGD in organotypic hippocampal slices where we could more carefully control the experimental conditions. Consistent with previous reports (Lushnikova et al., 2004), OGD caused a loss of hippocampal neurons, particularly in the CA1 subfield [**Figure 5A**; apoptotic cells labeled using propidium iodide (PI)]. We found that approximately 60% of CA1 were PI-positive at 72 h post-OGD (**Figure 5B**). To determine whether SPARC expression was regulated by OGD in a similar manner to MCAO, we performed IF and immunoblotting analysis on slices subjected to OGD. We found that SPARC protein was robustly increased at 48 h following OGD (**Figures 5C,D**).

**FIGURE 6:**
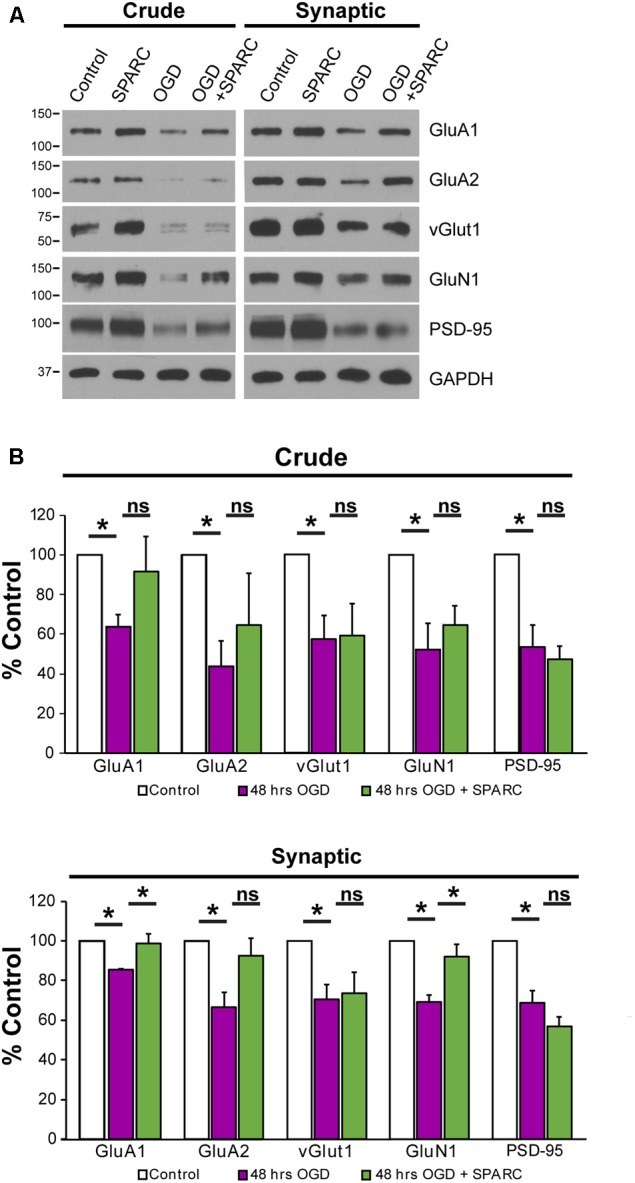
SPARC treatment rescues OGD-induced of loss of synaptic GluA1. **(A)** Representative immunoblots showing total (left panel, ‘crude’) and synaptic (right panel) proteins (GluA1, GluA2, vGlut1, GluN1, and PSD-95) from synaptosome preparations following control, SPARC (0.5 μg/ml, 48 h), OGD and OGD with SPARC treatment of organotypic slices. Slices were treated with SPARC 48 h prior to OGD and during the 48 h reoxygenation period. **(B)** OGD treatment of organotypic hippocampal slices induced a significant loss of synaptic proteins, however, SPARC treatment was able to rescue synaptic levels of GluA1 and GluN1 but not GluA2 [ANOVA with *post hoc* Holm–Sidak test (*n* = 4), ^∗^*p* < 0.05].

We next sought to investigate how SPARC might be regulating synaptic proteins during OGD. We prepared synaptosomes from slices subjected to OGD with and without SPARC treatment (SPARC treatment was for 48 h before and after OGD). OGD led to a significant loss of the synaptic proteins GluA1, GluA2, vGlut1, GluN1, PSD-95 (**Figure 6**), suggesting that OGD induces a loss of synapses, consistent with previous studies (Kovalenko et al., 2006; Nikonenko et al., 2009). Interestingly, pretreatment with SPARC prevented synaptic loss of GluA1 and GluN1 following OGD whereas vGlut1, GluA2 and PSD-95 levels were still reduced (**Figure 6B**). The rescue of synaptic AMPARs occurred even though overall levels of GluA1 were not significantly elevated, suggesting that SPARC preferentially promotes the recruitment and maintenance of GluA1-containing AMPAR complexes at synapses following OGD challenge rather than regulating overall GluA1 expression.

**FIGURE 7:**
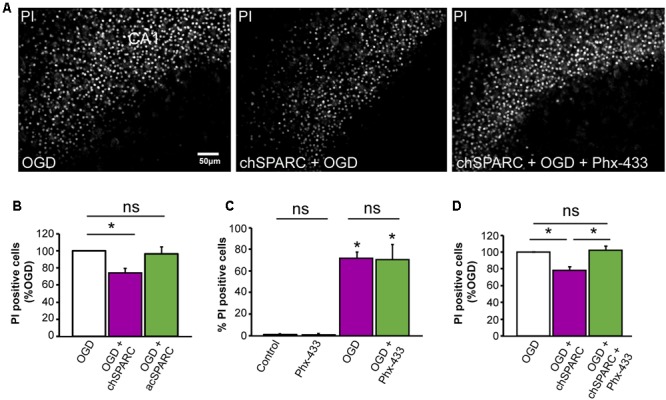
Protection of CA1 neurons from OGD-induced death by SPARC is mediated through Phx-433 sensitive GluA1-containing AMPARs. Organotypic hippocampal slices were exposed to oxygen and glucose deprivation (OGD) for 45 min followed by normoxic reoxgenation for 72 h. Percentage of apoptotic cells was assessed using propidium iodide (PI) labeling of apoptotic nuclei as shown in **(A)**. **(A,B)** Organotypic hippocampal slices were either pre-treated with SPARC (0.5 μg/ml for 48 h prior and following OGD in the reoxygenation period: chSPARC) before OGD or only acutely post OGD during the reoxygenation period (0.5 μg/ml: acSPARC). SPARC had a significant protective effect on CA1 neurons when slices were pretreated prior to OGD. **(C,D)** The protective effect of SPARC was inhibited by the AMPAR blocker Phx-433 (5 μM) **(A,D)**. Phx-433 alone had no effect on survival of CA1 neurons either in control or OGD conditions **(C)**. [ANOVA with *post hoc* Holm–Sidak test (*n* = 5), ^∗^*p* < 0.05]. Scale = 50 μm.

Given these findings, we investigated whether SPARC could affect the survival of CA1 neurons following OGD. To do this, we compared hippocampal slices subjected to OGD and acutely applied SPARC at time of OGD (acSPARC) or pre-treated with SPARC (chSPARC) for 48 h prior to OGD and following reoxygenation. We found that SPARC had a significant protective effect (∼26%) when slices were pre-treated with SPARC but not when it was acutely applied (**Figures 7A,B**). Interestingly, the protective effect of SPARC could be blocked by co-application of Phx-433 (**Figures 7A,D**). Phx-433 treatment alone had no effect on the number of apoptotic cells (**Figure 7C**). Together, these results suggest that SPARC regulation of Phx-433-sensitive GluA1-containing AMPARs at the synapse are important for improved cell survival and recovery following OGD.

## Discussion

In this study, we show that SPARC is upregulated in a model of ischemic stroke and following OGD and identify a novel role for SPARC in selectively regulating GluA1-containing Phx-433 sensitive AMPARs at hippocampal synapses. Furthermore, SPARC treatment increases GluA1-containing AMPARs at synapses of neurons exposed to ischemic injury and reduces the loss of CA1 neurons in hippocampal neurons following OGD. Our results suggest that SPARC and GluA1 cooperate to protect neurons in order to sustain neural connections following CNS injury.

Glial cells are involved in multiple phases of the ischemic cascade occurring following stroke, from the initial loss of blood flow and neuronal death, to the later processes of CNS repair and recovery (Gleichman and Carmichael, 2014). Both microglia and astrocytes respond to ischemic insult with microglia responding almost immediately post-injury and microglia and astrocyte proliferation peaking by day 3–4 post-infarct (Denes et al., 2007; Morrison and Filosa, 2013; Ding, 2014). Astrocytes also become reactive and increase their GFAP expression (Li et al., 2014; Choudhury and Ding, 2016), subsequently contributing to a glial scar (Becerra-Calixto and Cardona-Gomez, 2017). Interestingly, we observed that SPARC expression peaked at 3 days post MCAO, thus coinciding with the ‘peak’ period of reactivity (**Figure 4**). An increase in SPARC levels in microglia and astrocytes is consistent with previous studies using injury models, such as following a cortical stab wound (Mendis et al., 1998) or injury through deafferentation of hippocampal inputs (Liu X. et al., 2005). In addition, using a model of photo-thrombotic stroke, Lloyd-Burton et al. (2013) also found an increase in SPARC in activated, hypertrophic astrocytes in the peri-lesion region at 7 days following injury. However, they found that SPARC upregulation was selective to astrocytes and was in fact decreased in microglia surrounding the lesion. The reason for this difference is unclear, but perhaps it can be attributed to the difference between the two stroke models; for instance the microglial and inflammatory response resulting from photothrombotic stroke vs. MCAO may be different. In support of this, a recent study comparing the two stroke models found that the activation state and inflammatory profile of the microglia surrounding the stroke lesion were significantly different (Cotrina et al., 2017). Additionally, since Lloyd-Burton et al. (2013) found that there was an increase in microgliosis in SPARC-null mice following photothrombotic stroke, it would be interesting to further investigate the role of SPARC on microglia proliferation and inflammation in a MCAO model.

Interestingly, in all cases above, SPARC upregulation only occurred several days following injury, suggesting that SPARC is especially important during the processes of reactivity, and therefore may be important for the initiation of tissue repair and restoration of neural circuitry which occurs following initial loss of neurons (Carmichael, 2016). We found that exogenous SPARC was neuroprotective only when it was applied prior to ischemic insult (**Figure 7B**). This suggests that SPARC pre-treatment changes the state of the neurons, perhaps making them less sensitive to excitotoxic injury resulting from OGD. Our data shows that SPARC treatment of control neurons and hippocampal slices led to an upregulation of synaptic GluA1 levels (**Figures 1, 2**) and an increase in mEPSC amplitude mediated by GluA1-containing AMPARs (**Figure 3**). Furthermore, we observed that the neuroprotective effect of SPARC required Phx-433 sensitive AMPARs (**Figure 7**), most likely composed of GluA1-containing Ca^2+^-permeable AMPA subunits, since SPARC promoted the selective recovery of synaptic GluA1 (**Figure 6**) post-OGD. This increase in synaptic GluA1 levels and GluA1-GluA1 interactions by SPARC could be caused by mobilization of GluA1-containing AMPARs at postsynaptic sites or from extrasynaptic regions into the synapse and do not necessarily reflect an exchange of pre-existing AMPARs with differing subunit composition at synapses. In addition, our data does not rule out a possible contribution of GluA2-containing AMPARs in SPARC-mediated changes in synaptic strength and neuroprotection post-OGD. We observed a trend for a SPARC-induced increase in GluA2 expression (**Figure 2**) in control conditions and following OGD (**Figure 6**). In addition, in analyzing the kinetics of mEPSC events, we observed an overall increase in mEPSC decay time for all events upon SPARC treatment (Supplementary Figure 2D) and a decrease in decay time for small events (<10 pA) (Supplementary Figures 2C,D), perhaps due to SPARC-mediated changes to GluA1 homomeric and GluA2-containing AMPARs at synapses. Taken together, it is possible that the initial influx of calcium through homomeric GluA1-containing AMPARs may induce signaling pathways that facilitate the stabilization or expression of GluA2-containing AMPARs, which may then contribute to neuronal survival following ischemia as has been previously reported (Liu et al., 2004; Henley and Wilkinson, 2016).

We had previously observed that SPARC-deficient mice exhibited an increase in AMPARs and synaptic strength that was rescued upon application of recombinant SPARC (Jones et al., 2011). At first glance, this seemingly contrasts with our results here showing that SPARC can upregulate GluA1-containing AMPARs. The difference for the effect of SPARC in these two contexts may be explained by the ability of SPARC to function differently during development and following nervous system injury and in a concentration-dependent manner. Indeed, we found that an intermediate concentration of SPARC was most effective at regulating GluA1-containing AMPAR vs. low or very high concentrations (Supplementary Figure 1). We used a concentration of 0.5 μg/ml which represents an approximately 2.8-fold increase in soluble SPARC levels and interestingly is similar to the elevation in SPARC levels observed following OGD or MCAO (**Figures 4C, 5D**; approximately 2-fold) but which exceeds the concentration of that replaced in SPARC-deficient cultures previously (data not shown). Additionally, it is also important to note that in SPARC-deficient mice, SPARC is absent throughout development and synapses develop in the absence of SPARC leading to enhanced synaptic strength and defects in plasticity. Conversely, in this study, exogenous SPARC is added to wild-type cultures to mimic upregulation following injury or disease. It is conceivable that SPARC has differential effects of synaptic signaling pathways during development and following injury/disease.

The notion that Ca^2+^-permeable AMPARs can be protective in ischemia was surprising given that increased permeability to Ca^2+^ by downregulation of GluA2 is believed to lead to excitoxic cell death immediately following ischemia (Pellegrini-Giampietro et al., 1992, 1997; Oguro et al., 1999). Therefore, one might assume that an increase in Ca^2+^ through GluA1-containing, GluA2-lacking AMPAs would be detrimental for the tissue. However, it is important to note that Ca^2+^ permeability of AMPARs can also be conferred to GluA2 containing receptors if they are no longer edited at the Q/R site (Hume et al., 1991; Burnashev et al., 1992; Wright and Vissel, 2012). Furthermore, several recent studies have suggested that the contribution of GluA2-lacking AMPARs to excitotoxic-induced neuronal loss may not be as simple as initially predicted. Firstly, with currently available AMPAR antagonists, one cannot distinguish between GluA2-lacking and unedited GluA2-containing Ca^2+^-permeable AMPARs. Secondly, whilst there is a decrease in GluA2 mRNA and protein levels in the hippocampus following ischemia (Gorter et al., 1997; Noh et al., 2005; Dixon et al., 2009) as well as GluA2 internalization (Blanco-Suarez and Hanley, 2014), an concomitant increase in GluA1 was not reported (Pellegrini-Giampietro et al., 1992; Soundarapandian et al., 2005). Conversely, it has been demonstrated that the increase in Ca^2+^-permeable AMPARs following ischemic insult may also arise due to an increase in unedited GluA2-containing AMPARs and downregulation of ADAR2, the nuclear enzyme responsible for Q/R editing (Peng et al., 2006; Filippini et al., 2016). Indeed, overexpression of unedited GluA2 (Q) in dentate granule cells conferred vulnerability of these neurons to excitotoxic death (Liu et al., 2004), whilst restoration of ADAR2 levels in CA1 protected neurons from ischemia (Peng et al., 2006). Thirdly, there is strong evidence for a role for GluA1 in dendritic development and growth. A role for GluA1 in mediating dendritic growth and arborisation in motor neurons via SAP97 was demonstrated (Inglis et al., 2002; Prithviraj et al., 2008; Zhou et al., 2008). Furthermore, cortical neurons overexpressing GluA1 show increased dendritic complexity and length (Chen et al., 2009), whereas loss of AMPA transmission via GluA1 in Xenopus optic tectal neurons reduced dendritic arbor growth (Haas et al., 2006). Finally, GluA1-containing, Ca^2+^-permeable AMPARs have an established and important role in the induction of forebrain plasticity during development, following experience and in disease (Caleo, 2015; Henley and Wilkinson, 2016). Taken together, one possibility is that increased GluA1 induced by SPARC could serve to stimulate circuitry re-wiring and replace connectivity through regulation of dendritic growth and plasticity through Ca^2+^-permeable AMPARs (Murphy and Corbett, 2009; Nudo, 2013 for reviews on plasticity following stroke). In future studies it would be interesting to test the effect of increased SPARC on dendrite and synapse reorganization. Furthermore, since SPARC is a secreted protein, it would be important to understand the relative contributions of SPARC from microglia and astrocytes during the injury response, and whether secretion occurs locally to regulate adjacent synapses.

## Ethics Statement

This study was carried out in accordance with the recommendations of the Canadian Council for Animal Care. The protocol was approved by the Montreal General Hospital Facility Animal Care Committee.

## Author Contributions

EJ, YB, JZ, and SC performed the experiments and analyzed the data. EJ and KM conceived the study, designed the experiments and wrote the manuscript with contributions from YB and JZ.

## Conflict of Interest Statement

The authors declare that the research was conducted in the absence of any commercial or financial relationships that could be construed as a potential conflict of interest.
