# Supplementary figures and images for: SPARC and GluA1-Containing AMPA Receptors Promote Neuronal Health Following CNS Injury
Source: Front Cell Neurosci. 2018 Feb 1;12:22. doi: 10.3389/fncel.2018.00022 (PMC5799273; doi:10.3389/fncel.2018.00022)

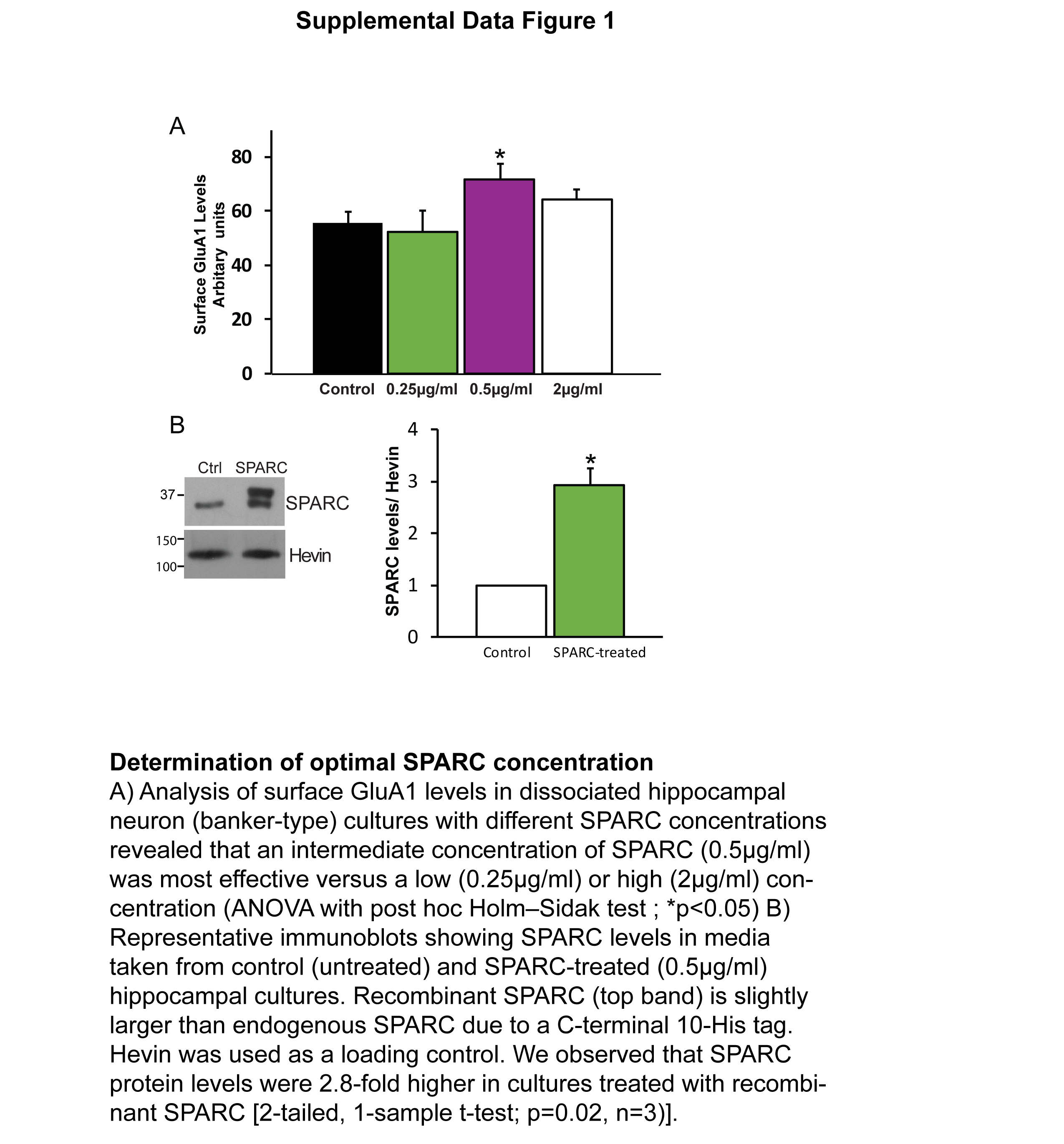

Supplement: Supplementary file 1 [file Image_1.TIF]

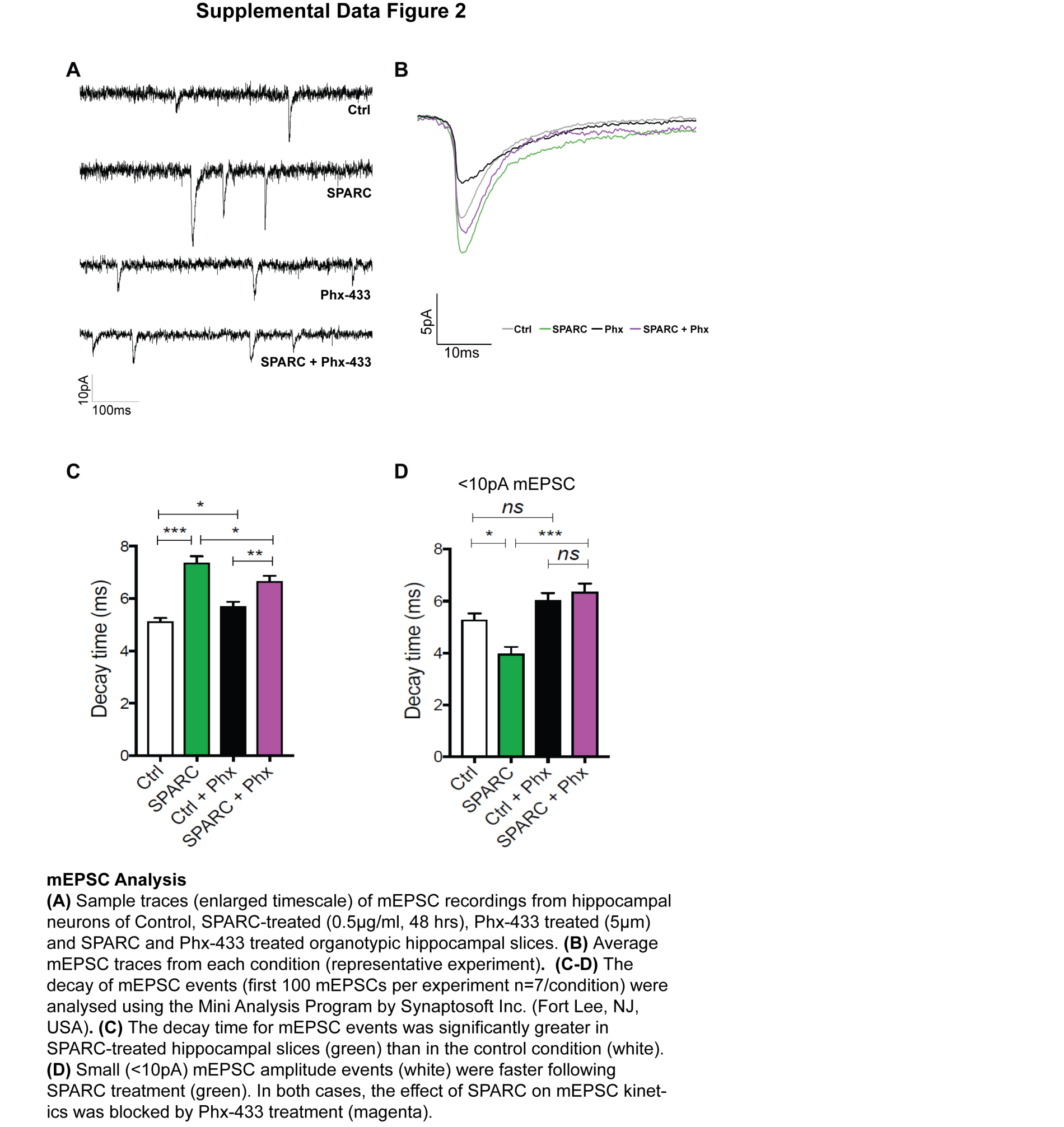

Supplement: Supplementary file 2 [file Image_2.TIF]
